# Printability of Bioinks: A Consolidated Definition for Additive Manufacturing

**DOI:** 10.1021/acsomega.5c00727

**Published:** 2025-11-26

**Authors:** Matheus A. A. Resende, Eliana C. S. Rigo, Andrés Vercik

**Affiliations:** † Interunit Graduate Program in Bioengineering (PPGIB), 28133 University of São Paulo, 13566-590 São Carlos, SP, Brazil; ‡ Department of Basic Sciences of Faculty of Animal Science and Food Engineering (FZEA), 146420 University of São Paulo, 13635-900 Pirassununga, SP, Brazil

## Abstract

Printability is a key concept extensively used in the context of bioinks for additive manufacturing. However, the absence of a universally accepted definition has led to inconsistencies in its application across studies. Some researchers use the term interchangeably with shape fidelity or shape accuracy, while others incorporate multiple aspects, including rheological properties and extrudability, into its definition. In some cases, the omission of cell viability further limits the scope of current research. As a result, studies that connect rheological properties to printability or employ physical and mathematical models to predict outcomes often fail to capture the complete manufacturing process due to the lack of definitional consistency. This review examines literature from 2011 to 2024, identifying key thematic clusters that contribute to a more robust understanding of printability. We propose an integrative definition that encompasses not only rheological properties across all stages of production but also geometry, shape fidelity, shape accuracy, and functionality—with particular emphasis on cell viability. This broader definition aims to foster greater consensus and guide future applications of 3D printing in bioprinting and other additive manufacturing fields.

## Graphical abstract

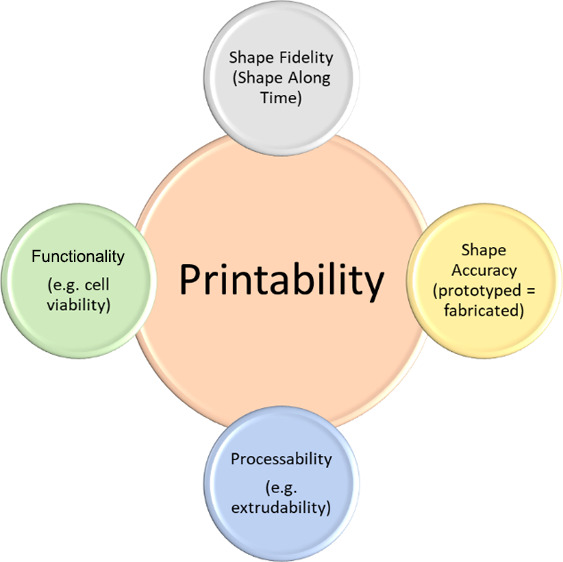

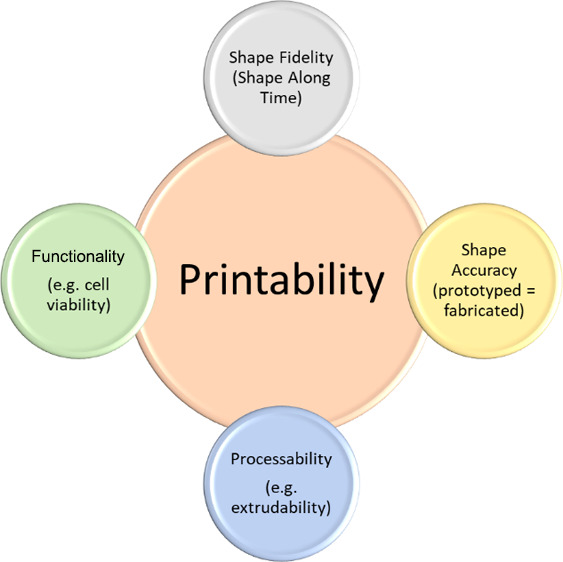

## 1 Introduction

Three-dimensional (3D) bioprinting is gaining significant importance in the fields of engineering and materials, particularly in the creation of synthetic and natural tissues using bioinks as raw materials.^1^ Currently, the global bioink market is estimated to be around 1.6 billion, with a forecasted increase of up to 20% of this value by 2030.^2^ This trend is driven by extensive research and development in several fields, such as regenerative medicine, drug production, and the printing of food components.^3,4^

To improve the printing process, there is a need not only to assess its quality under different conditions, materials, and applications but also to relate (understand standards parameters in the) the features of the printed object to material and process parameters. A concept extensively used for this purpose is printability. According to Naghieh et al. and Chen et al.^5,6^ printability is “the capability to form and maintain reproducible 3D scaffolds from bioink using the bioprinting technique.” Several studies have addressed the issue of predicting printability from material and/or process parameters in various contexts, including bioprinting,^5,7–10^ foodprinting,^11^ metallic inks,^12,13^ construction,^14,15^ conductive hydrogels,^15^ and other biomaterials.^16^

Ma et al.^17^ used rheological data and image processing to model extrusion capabilities of complex food materials for 3D printing which can be understood as printability. Elbadawi et al.^18^ conducted a study on the printability of drug delivery devices, using machine learning (ML) tools to predict dissolution properties based on material rheological data. Schwab et al.^10^ discuss the rheological aspects affecting the printability and shape fidelity of extrudable bioinks, highlighting the impact of shear thinning, viscoelasticity, and yield stress on printability. They also examine the effects of cell presence on the rheological properties of bioinks. Kyle et al.^1^ reviewed the concept of printability, emphasizing its importance for bioprinting, particularly for cost-effective modeling and process tuning.

Despite these studies and others, it is evident that a general consensus among researchers in the 3D bioprinting and printing field is still lacking.^1^ The need of a widely accepted definition of the concept of printability is not merely formal, but of fundamental importance in order to unify the meaning of a target property of a considerable number of studies. In other words, it is impossible to compare studies of apparently the same property, which means different things for different researchers. For instance, the biofabrication window paradigm, mentioned by Kyle et al.^1^ relating biocompatibility and printability is nonsense if the latter is not defined properly, leading to misinterpretation of results. Another issue refers to the attempts of quantify the printability parameter. Ouyang et al.^8^ have used a mathematical formula for printability based on a square:

$$P_{r}=\frac{\pi }{4}\frac{1}{C}=\frac{L^{2}}{16A}$$ 1

where *L* is the perimeter *A* is the area and *C* the circularity, given by

$$c=\frac{4\pi A}{L^{2}}$$ 2

Thus, for a square shape, Pr = 1, with lower values for more circular shapes (with a minimum value of 1/4) and higher values for other types of deformations. According to this point of view, printability is a purely geometric parameter—something controversial, as discussed in the next section—that essentially quantifies shape fidelity and/or shape accuracy. Moreover, these two terms “shape fidelity” and “shape accuracy” can be interchangeable for some authors, whereas they can mean different things for others. For instance, Schwab et al.^10^ state that “shape fidelity can be used to describe the shape retention of single filaments upon extrusion as well as of the printed construct as a whole compared to the original computer design and is sometimes referred to as print accuracy”, endorsing the understanding that all three terms are synonyms. Kyle et al.^1^ also consider printing geometry accuracy and shape fidelity to be synonyms. Fisch et al.^19^ addressed the accuracy and precision of bioprinting. Both concepts remain confused in that work. According to the authors, “Accuracy was determined based on the percentage difference between the mean of the measured extruded volume and the targeted volume and precision was determined based on the standard error”. In both cases, the measure refers to geometrical similarity. Gillispie et al.^20^ consider that shape fidelity is “shape fidelity is the ability of a bioink to maintain its shape upon deposition”, a vague concept as pointed out by themselves. On the other hand, printing accuracy “can be thought of as the degree to which printed filaments, features, and constructs match their intended size, shape, and location with respect to the printing parameters used”. In this sense, according to these authors shape fidelity refers to individual filament behavior whereas print accuracy indicates printed object quality or similarity (to desired geometry in genera CAD-produced). However, in that work, the authors state that “The most quantitative measure of printing accuracy is the width of extruded filaments”. So apparently, both concepts are related to the shape of filaments. However, later in that work, it is mentioned that “Experiments which evaluate printing accuracy utilized a single bioink and varied the print parameters. Studies which evaluate shape fidelity, meanwhile, compared their results among various bioinks”. Ma et al.^17^ equal printability to extrudability, whereas Temirel et al.^21^ consider printability and shape fidelity as different properties (even they can be somehow related). Therefore, several terms like ″shape fidelity″, ″shape accuracy″, “printing precision”, among others, are found in literature with vague or ambiguous definitions, which many times are used interchangeably—including the printability itself—and inconsistently. This lack of formal definition might lead to confusion or even misinterpretations of results in studies of printability.

Given the significant role of printability in characterizing the printing process, a universally accepted definition is essential, particularly in the context of developing potential standards for 3D printing and bioprinting.^1^ In this study, we systematically reviewed the literature using text mining tools to analyze the varying interpretations of printability. After this revision, a tentative yet comprehensive and broad definition of printability is proposed and intended to be valid across diverse fields and applications of 3D printing, including bioprinting. Concepts such as shape fidelity and shape accuracy, among others, must be clearly and consistently defined. These observations highlight that printability is not only a contentious topic but also inherently multidisciplinary. Therefore, this work provides a detailed review of the concept of printability with a focus on bioinks as described by Copus et al.^22^ It examines the implications of different definitions, the key factors considered in each case, which lead to the proposed and broader definition applicable to the various scientific, technological, and industrial contexts where additive manufacturing is employed.

Additionally, an important observation of this work is that cell viability should be included as a key aspect of printability in the proposed definition. This inclusion is important because large-scale production tends to be more dynamic, and the concept of printability may extend to a parameter that relates to the entire production process.^8,23^ Since the capacity for metabolic function in a bioink is essential, the definition of printability should adopt this broader perspective. Thus, the systematically review of the existing literature presented here traces the evolution of printability and finally proposes a comprehensive definition that encompasses rheological characteristics geometric properties, and key biological aspects, such as cell viability.

## 2 Methods

### 2.1 Bibliographic Review

The articles included in this review were selected based on their relevance, identified through searches on the ScienceDirect and Google Scholar platforms over the past 12 years, with additional consideration given to the impact factor of the publishing journals. The keywords used in the search were ″Printability,″ ″Printability Definition,″ ″Printability and Bioprinting,″ and ″Printability Definition in Bioinks”. Alongside a brief review of the significance of printability and the rheological and geometric properties of bioinks, the selected articles also implicitly contributed to defining the term ″printability.″

For clarity in analyzing and presenting the results, the studies framework was categorized into specific topics, according to their theoretical framework and the definition of printability considered. The primary studies identified through the search are discussed within the text, and additional reviewed articles are summarized in Table 5. This review was conducted using a text mining tool, to implement a methodology adapted from Patel et al.,^24^ employing Orange, an open-source software environment with data science and machine learning resources. In their study, Patel et al. discuss how text mining tools used for clinical trial searches can be extended to other fields. Based on this concept, we applied text mining techniques to gather and process textual data, referred to as a corpus, ultimately performing a structured text analysis.

The research process in Orange followed a systematic workflow. The first step involved data retrieval, in which relevant articles were identified by searching for predefined keywords across the platforms. The selected references were exported in .bib format and subsequently converted to a .csv file. This conversion facilitated the structuring of bibliographic data into a tabular format, allowing for further analysis in worksheets.

Next, within the Orange interface, the.csv file was imported into the Corpus widget, which categorizes articles based on keywords. The text was then preprocessed to filter relevant topics, followed by visualization using the Topic Modeling widget. The next stage involved selecting relevant articles based on predefined criteria and analyzing article distribution through Multidimensional Scaling (MDS). The structure of the Orange workflow and the results from the MDS correlation analysis score of those articles are show in Figure 1. Additionally, Figure 2 presents a mind map generated by the software, displaying the most frequently occurring keywords in the reviewed articles. The term ″Printability″ is centrally positioned as the most cited keyword, with related terms surrounding it, emphasizing their relevance to the review study.

**Figure 1:**
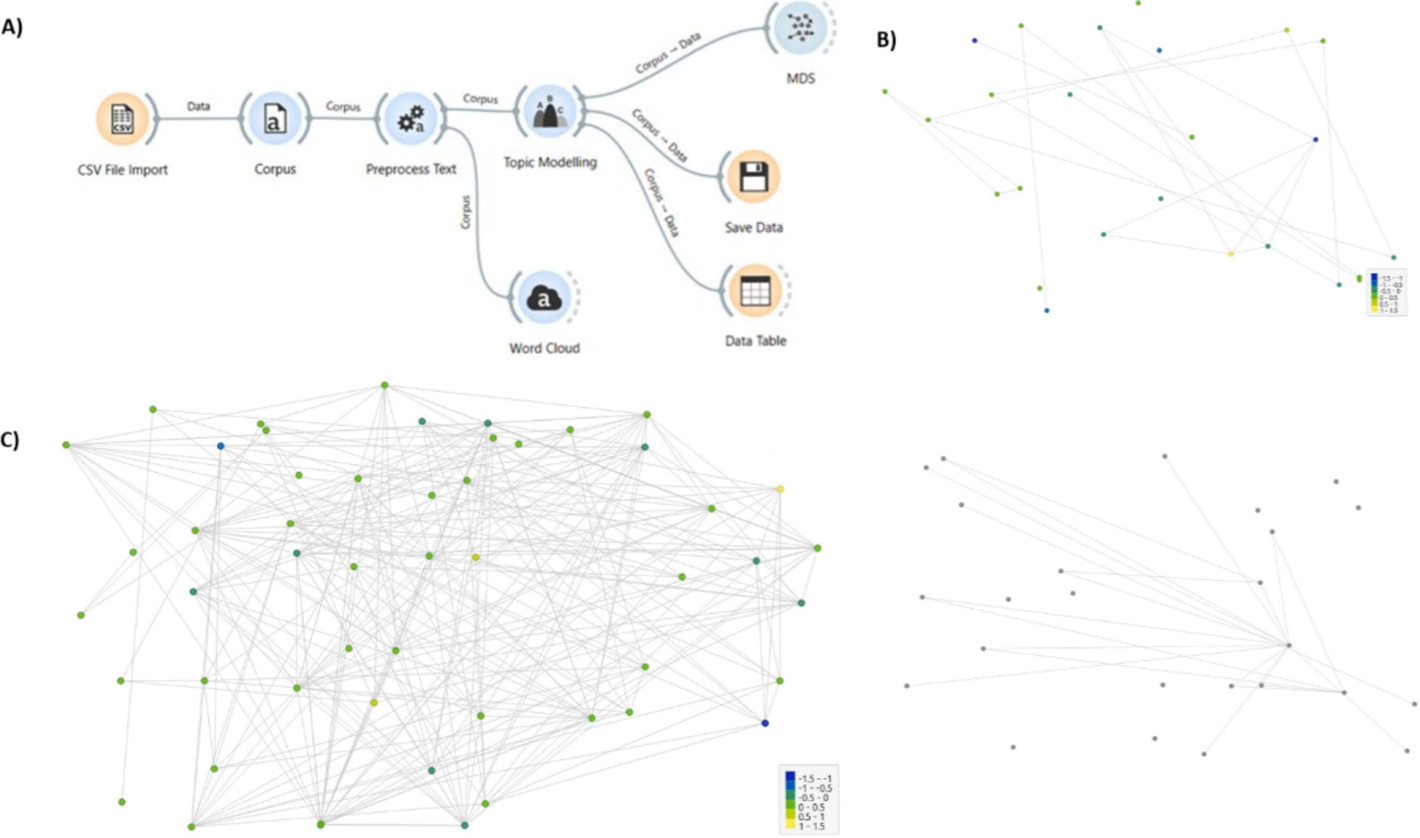
(A) Workflow of the Orange Software for text mining of articles. Keywords were selected based on the criteria outlined in Table 1. (B) Since the platforms do not export all search references at once, multiple iterations were necessary, retrieving 25 articles per batch. The MDS results ranked articles based on thematic similarity until the final selection for Table 5. At the top, articles with higher similarity are grouped, while at the bottom, less similar articles—those that were immediately discarded—are shown. (C) MDS results for the articles included in the review, as listed in Table 5.

**Figure 2:**
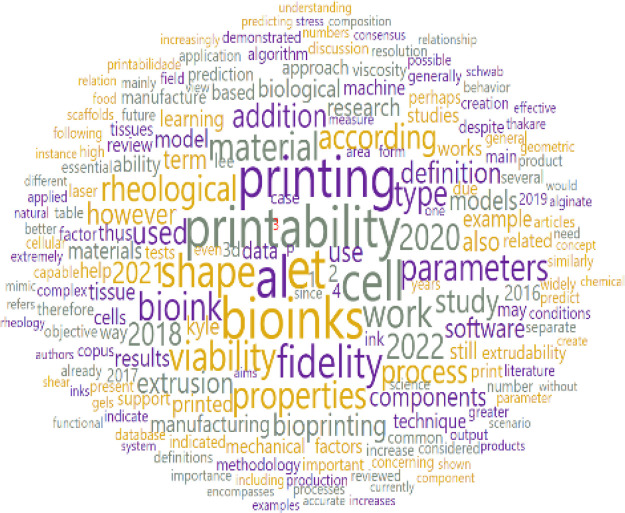
Representation of a mind map developed by software featuring keywords from the reviewed articles. It is evident that ″printability″ is at the center as the most mentioned word, and surrounding it are important terms related to the study of the review.

At the beginning of the search, 17,109 results were found with the word ″printability.″ Articles that do not explicitly specify the word ″printability″ but emphasize the importance of factors related to it, such as shape fidelity and cell viability, were also considered. Table 1 demonstrates how the number of results decreases according to the specification of terms related to printability.

**Table 1:** Search Results

keyword (s)	search results
printability	17.109
printability definition	3.566
printability and bioprinting	1.736
printability and bioinks	1.375
definition printability in bioinks	226
used	52

The results indicate that printability is referenced as a concept mainly related to rheological properties, extrudability capacity, the geometry of the print, its support after printing, and the viability of the print for cell growth and other metabolic processes. Depending on the research objectives, these parameters may vary or even be ignored. Additionally, they may be associated but separately, e.g., not included in the definition, according to the expressions used in the bibliography. The following are the results of the literature review according to the topics of the mentioned parameters.

## 3 Preliminary Assessment about Printability

Additive manufacturing, including 3D bioprinting, has emerged as a pivotal technology for fabricating complex structures directly from digital models. However, as bioprinting scales toward industrial and clinical applications, a deeper understanding of material behavior under printing conditions becomes essential to ensure both the reproducibility and functionality of bioinks.^25^

Printability is influenced by the rheological properties of bioinks, including viscosity, shear-thinning behavior, and yield stress, which govern the ease of extrusion and structural stability during printing. Extrusion-based techniques, the most common bioprinting methods, require careful balancing of these parameters to achieve precise geometries without compromising cell viability. Therefore, rheological characterization plays a fundamental role in assessing how bioinks behave under flow and recover postextrusion.^26^

Rheology governs the processability of the inks (extrudability) and ultimately the properties of the constructs, whose similarity to the original digital model must be guaranteed, as well as their functional properties, depending on the aimed application. Shape accuracy relates to the match between the virtual design of the model and the printed object, while shape fidelity ensures that the printed structures maintain the desired shape and dimensions throughout the process, from extrusion to postprinting stages.^10,27^ Another important factor is cell viability, since bioinks contain living cells. Ensuring that cells survive and proliferate within the printed structure directly affects the performance and long-term success of bioprinted constructs.^25^

The following section aims to explore the intricate relationship between material properties, geometric aspects, and cell viability in the context of printability. A comprehensive understanding of these factors is crucial for developing bioinks and protocols that ensure the seamless integration of bioprinting into manufacturing workflows, advancing the potential for research and industrial applications.

### 3.1 Reology

#### 3.1.1 Rheological Parameters

The initial step in understanding the behavior of materials used in bioprinting is the study of rheology.^1^ Rheology can be defined as the ″study of the deformation and flow of matter.^21^ This field emerged from observations of the behavior of real materials, which deviated from the ideal behavior of solids and liquids described by classical mechanics. These deviations require the interpretation of material parameters such as elasticity and viscosity. Elasticity is the ability of a material to return to its original conformation after the removal of an external force. Viscosity, on the other hand, quantifies the energy dissipation within a fluid due to resistance to the movement of its constituent particles relative to each other, often conceptualized as internal friction. The viscosity of a material is typically measured by examining the relationship between shear stress and shear rate when forces are applied in a manner analogous to a scissor cut.^23^

With advancements in measurement equipment such as viscometers, extruders, and rheometers, more experimental modalities were developed, such as rotational tests. In this case, the determination of the viscoelastic properties can be interpreted in terms of two variables, *G*′ which represents the capacity to store potential for the material to resist deformation (elasticity), and *G*″, which indicates the lost potential of the material for deformation, which causes the material to flow (viscosity). This quantitative approach is crucial for process standardization in large-scale production, particularly in additive manufacturing. Additionally, the methods used to identify these parameters are essential for understanding printability.^23^ Table 2 provides a concise summary of the equations used to determine rheological parameters in some studies, along with the methods employed to measure these quantitative parameters.

**Table 2:** Understanding the Concept of Printability in Bioinks Requires the Definition and Examination of Key Rheological Parameters^a^ ^,^ ^b^

assay	equipment example	rheological parameter	definition	reference
parallel plate/oscillatory	CSL2 Rheometer	viscosity	general Newton’s law: $\tau =\mu \frac{dV}{dt}$	Bicerano et al.,^28^ Chen et al., 2018.
			viscosity of polymers $[\eta ]=\frac{\eta (\mathrm{dispersion})}{\eta (\mathrm{fluid})}$	
parallel plate	MCR301 Rheometer	shear rate	γ̇= $\frac{v}{h}$	Jadhawa et al., 2018; Paxton et al.^9^
parallel plate	ARES-LS2 Rheometer	shear ticking	$\eta ={\left[1+\left(\lambda {\mathrm{\gamma ̇}}^{2}\right)\right]}^{n-1/n}$	Habib et al.,^86^ Islam et al., 2021; Vergne, 2017.
rotational	Modified Rotational Pump	shear yield stress	τ = τ_0_ + *a*γ̇*b*	Mandal et al.,^26^
			H–B model for shear-thickening materials	

a These parameters can be quantitatively measured through various techniques. In the context of additive manufacturing, it is crucial to evaluate the relevance of these methods and the equipment employed for accurate assessment of printability. Also, the mathematical models can provide a good approach to measuring printability in some cases, as they can relate various parameters in an equation.

b τ = tension; d*V*/d*t* = shear velocity; [η] = apparent viscosity; (γ̇) = shear rate; *v* = velocity; *h* = distance of the plaques; η = viscosity; μ = shear viscosity; λ = characteristic time; *n* = power law exponent; *a* = consistent factor; *b* = flow index.

Yin et al.^100^ provide a table with additional parameters calculated from those listed above. Rheological standards are further supported by mathematical models, as demonstrated in the research by Paxton et al.,^9^ which quantitatively determines shear rates through viscosity calculations. Copus et al.^22^ also enumerate several other rheological parameters, such as filament length, extrusion forces, and porosity, which can be crucial for assessing the printability of bioinks. These parameters are essential for developing a more comprehensive understanding of printability.

The interpretation of rheological results depends significantly on the type of equipment used. This study primarily focuses on extrusion techniques, as they are the most commonly employed methods for analyzing materials.^1^ Advanced measurement techniques, such as laser-assisted equipment, can provide higher accuracy but are associated with substantially higher costs compared to extrusion methods. The selection of equipment for measuring rheological properties is influenced by several factors, including financial constraints, material composition, and specific research objectives.^28,29^

It is important to note that cells or biological components in bioinks can alter the mechanical properties of the material due to changes in forces such as surface tension, which affect viscosity and elasticity.^30^ Consequently, this can influence the printability of the ink and must be included in its definition. According to Lee et al.,^15^ printability is directly associated with the rheological properties of bioinks. Their study demonstrated that increasing the concentration of components, such as collagen, enhances the material’s elasticity without the need for chemical treatments. In extrusion-based printing, this is also linked to extrudability—a parameter that reflects the material’s ability to undergo extrusion through microscale dimensions, such as the tip of a needle—an essential step in the final stages of the printing process.

As the complexity of a study increases, additional measurements or calculations may be required to better elucidate the rheological characteristics of a fluid. This complexity is often amplified by the presence of various polymers and biological residues in the fluid.^1^ In the context of additive manufacturing, achieving accurate rheological data is critical, underscoring the importance of employing robust data management tools.^9^ Ma et al.^17^ highlight this approach in developing a model for analyzing the rheological and imaging data of complexes used in the food industry.

These considerations emphasize the intrinsic link between rheology and the successful printing of bioinks.

### 3.2 Main Bioprinting Techniques

The primary objective of using bioinks in additive manufacturing is to facilitate the large-scale production of parts and objects with constituents that closely mimic human body tissues through the selection of an appropriate printing technique.^1^

The following section provides a concise overview of the main techniques for bioink printing/bioprinting, accompanied by a brief discussion of the advantages and disadvantages of each method:

- Extrusion Printing: This technique involves passing a polymer filament through a nozzle, depositing it onto a platform where it solidifies into a predefined geometry.^25^ This method offers the advantage of printing materials with high viscosity while maintaining high fidelity to the intended geometric shape. However, limitations include low printing speed and challenges associated with the gelatinization time of the samples. Extrusion printing is widely employed in the fabrication of filaments for various tissues, including bioinks for blood vessels, cartilage, heart valves, and nervous system tissues.^1^
- Stereolithography Printing: According to Skardal et al.,^31^ this technique uses ultraviolet light to assist in printing photopolymers. This light solidifies the polymer layer by layer. Despite providing good print resolution and increasing the production speed of bioinks, this technique is limited to photosensitive materials and may pose an issue in printing biological materials such as DNA and RNA due to the use of ultraviolet radiation. Applications of this technique include printing components of blood vessel tissues and organs like the liver, heart, and skin. It is also used for preparing inks with electrical components, such as chips.^1^
- Laser-Assisted Printing: This technique utilizes a laser as the energy source to print bioinks onto metallic receptors, along with a tape containing the substrate of cells or cellular structures to be fabricated, referred to as the receptor substrate. The method provides high printing resolution and minimizes mechanical stress on the polymers of the printed structure. However, it is associated with high costs, and the heat generated by the laser can potentially damage the cells in the bioink, requiring continuous calibration and monitoring of cellular activity. Applications of this technique include the fabrication of adipose tissue, skin, and blood vessels.^1^

Despite the existence of other methods for fabricating objects using bioinks, the above-mentioned methods are emphasized due to their significance in market research and research and development within this field.

### 3.3 Bioinks

The use of bioinks is the factor that characterizes the 3D printing as bioprinting.^11^ It is important to classify these materials based on their intended applications, which can be divided into several categories, such as support bioinks, transition bioinks, structural bioinks, and functional bioinks.^32^ This classification is summarized in Table 3.

**Table 3:** Types of Bioinks^a^

name	definition
support bioink	bioinks used to support cell populations, acting as a cellular matrix
transition bioinks	consists of bioinks of temporary materials that can be easily removed to form internal hollow channels within a printed structure
structural bioinks	bioinks that provide support for the structural integrity of a print
functional bioinks	bioinks that provide biochemical, mechanical, or electrical functions to influence tissue behavior

a Groll et al. (2016).

According to Gungor-Ozkerim et al.,^33^ an effective bioink must possess rheological, chemical, and biological properties that mimic or even replicate those of the original biological tissue. Specifically, a bioink should exhibit appropriate stiffness and plasticity in its geometric conformation to provide structural support while facilitating the necessary chemical exchanges within the cellular matrix environment, aligning with the morphological functions required for the intended implementation.

To achieve these properties, bioinks are commonly formulated from mixtures of natural polymers, such as alginate, protein-based gelatins, and collagens, with addition of other components. These formulations also incorporate cell products or living cells, depending on the specific requirements of the application.^32^

In this work, bioink is defined as ″Printed materials designed primarily to deliver cellular contents or cells, ensuring their biological activity upon application to the targeted tissue.”^34^

Regarding printability, the chemical bonds of the components in bioinks, whether polymer chains or of another nature, clearly influence their overall properties. The presence of cells or biological components adds complexity to the study of this specific type of material.

### 3.4 Shape Fidelity and Shape Accuracy

The terms ″Shape fidelity″ and ″Shape accuracy″ are controversial in the literature, since they appear as synonyms in some works, whereas they refer to different properties in others. In those cases, it is preferable to consider these parameters separately.^1,35^ This confusion may arise from the similar meanings of the terms ″fidelity″ and ″accuracy.″ It may be more appropriate to use distinct concepts—for instance, shape accuracy to describe reproducibility relative to the CAD design, and shape fidelity to refer to the maintenance of the shape over time.^35^

The concept of shape accuracy is generally related to the precision of a printed material compared to a predicted reference, such as a computer-aided design (CAD) drawing. This can pose a challenge in determining rheological parameters that influence this property during the manufacturing process.^119^ In some studies, utilizing 3D printing, like Thakare et al.,^27^ shape accuracy is associated with comparing the dimensions of the printed material’s scaffolds with values predetermined by CAD. Thus, the more print/design similarity, higher shape accuracy. Another important factor highlighted by this study is the material’s ability to maintain its shape–shape fidelity-in a way that does not compromise the cellular metabolism within the bioink.

Lee et al.^15^ argue that all these features depend on the component concentration of the bioink, and should be analyzed in order to evaluate or predict possible changes in the rheological parameters. However, it is emphasized that the rheology-shape fidelity relationship depends on several factors, which might be better understood before having a predictive tool for the bioink behavior after extrusion.^36^ For example, understanding the response of the bioink under shear stress that exhibits shear thinning (decrease in viscosity with the application of stress) during the extrusion phase, can provide better print properties (shape fidelity and/or accuracy) while retaining the extrudability.^37^ Typically, shape fidelity is quantified by measuring the dimensions of a printed piece or some dimension of the printed material, such as the width and/or height of a single filament after extrusion.^10^

Copus et al.^22^ and Lee et al.^15^ suggest that gels are the most suitable materials for bioinks, as their mechanical properties align with rheological patterns that closely mimic biological tissues. Additionally, as mentioned earlier, mathematical models can be employed to describe those geometric parameters. For instance, Schwab et al.^10^ demonstrated that shape fidelity can be predicted for extrusion under continuous pressure, and the bioink filament after printing can be analyzed.

Therefore, taking these points into consideration, this work defines shape accuracy, as ″the retention of the printed material’s shape compared to a predicted reference.″^27^ And shape fidelity as “The capacity of the printed object to maintain its geometric form for a specified period following the printing process”.^1,27,35^

For printability, it is noted that many studies define printability primarily based on geometric standards, including shape fidelity and shape accuracy. While this approach is reasonable in many cases, when considering a broad approach, the concept of printability solely as a geometric criterion can lead to an oversight of print performance. Therefore, a comprehensive definition should include not only geometric parameters but also cell viability to ensure efficiency in large-scale production.

### 3.5 Cell Viability

With the growing use of biological components in 3D printing and the application of printed materials in biological systems, the need to evaluate the biological functionality of these materials for clinical testing has become increasingly important. This functionality is often assessed through cell viability.^1^

For a bioink to exhibit good results, it must possess both biocompatibility and the ability to support cellular growth within its structure.^22^ On the other hand, preserving the biological properties of the ink throughout the manufacturing process introduces significant complexity. Cellular viability is critical, as it is associated with the survival of cells or cellular components of interest and the prevention of contamination by undesirable structures that could increase the material’s cytotoxicity. Consequently, two common strategies in bioprinting have emerged: extrusion with pre-existing cultures or cellular components, and the addition of these components postprinting, such as in the case of scaffolds.^1^ It is well recognized that the introduction of chemical or physical processes aimed at ensuring cellular viability can alter the overall behavior and quality of a bioink, making cellular viability a crucial parameter for assessing printability.^22^

An illustrative example is provided by Lee et al.,^15^ who found that the concentration of natural collagen in bioink—a component used to support cells after the extrusion process—also enhanced the elasticity of the final product. Conversely, Quílez et al.^38^ explored the impact of components such as penicillin, an antimicrobial agent used in cell cultures, on the rheological properties of bioinks. This information could be vital for additive manufacturing regarding bioink printability. In light of this discussion, the present work adopts the interpretation of cellular viability as ″the capacity for the reproduction and/or biological functionality of cellular components in printed materials.″^27^

### 3.6 Some Definitions of Printability Found in the Literature

Several definitions of printability can be found in current literature addressing bioinks. It is important to highlight that there is no consensus on whether these definitions are incorrect, as each study focuses on issues related to the objectives of the research. Later in section Further Topics, we demonstrated the need of a standardized definition of printability regarding additive manufacturing.^1^

According to Copus et al.^22^ printability, in its broadest sense, refers to a material’s capacity to be printed. The same study provides a more formal definition: ″The ability of a material, when subjected to a specific set of printing conditions, to be printed in a way that results in printed objects suitable for a particular application″ (Copus et al., 2022, p. 154). Thus, printability encompasses several factors, including extrudability, which is the material’s capacity to be extruded through a printer nozzle at a micrometer scale; shape fidelity, which pertains to the material’s ability to maintain the virtual object’s geometry after printing; and shape precision, which relates to the ink’s capacity to maintain tissue-mimicking conditions despite environmental changes. Consequently, printability integrates these aspects and can be manipulated based on the printing objectives.

Li et al.^39^ used an interesting strategy to define the printability of starch- and surimi-based biomaterials by expressing it as the sum of the products of key rheological parameters obtained from rheometer tests. Since the objective of these materials is not related to cell growth and metabolism, no cell functionality parameters are included in this approach. Other studies have adopted a similar approach, representing printability through either equations or written definitions. These parameters are detailed in Table 4.

**Table 4:** Some Definitions of Printability^a^

definition	equation	authors
alignment between manufacturing suitability and the ability to encapsulate cells, along with the capacity to maintain the viability of cellular structures.	none	Kyle et al., 2018
parameters based on shape fidelity, biocompatibility, resolution, and cell support.		Lee et al., 2020
without a precise definition, it notes that the printability of a product is related to its rheological properties.	none	Elbadawi et al., 2020
difference in quantitative and qualitative parameters between projected printing and experimental printing.	none	Fu et al., 2021
printability as a coefficient that represents the sum or the product of rheological indicators, resulting in a printability score	*Y* = (*a*_1_)*a* + (*b*_1_)*b* + (*c*_1_)*c* + (*d*_1_)*d* + (*e*_1_)*e* + (*k*_1_)*k* + (*n*_1_)*n* + τ_*y*_^a^ ^b^	Li et al. (2022)
	$P_{r}=\frac{\pi }{4}\frac{1}{C}=\frac{L^{2}}{16A}$ ^c^	Ouyang et al.^8^

a It is evident that printability encompasses various defining parameters, including, in the case of Li et al. (2022), where it is proposed in the form of an equation.

b *Y* = printability coefficient; *a*_1_, *b*_1_, *c*_1_, *d*_1_ *e*_1_, *k*_1_, *n*_1_ = constants obtained by the rheological experiment; *a* = hardness; *b* = elasticity; *c* = viscosity; *d* = reversibility; *e* = gel strength; *k* = viscosity coefficient; *n* = power law index; τ_y_ = yield stress.

c *L* is the perimeter, *A* is the area, and *c* is the circularity, given by $c=\frac{4\pi A}{L^{2}}$ .

Kyle et al.^1^ note that, although the term printability is crucial for 3D bioprinting, a precise definition correlating the rheological parameters of bioinks—such as viscosity, extrusion, and gelation—with cell viability properties like bioactivity, encapsulation capacity, and shape fidelity is lacking, reinforcing the discussion about the terminology.^15^

Lee et al.^15^ also emphasize the need for a clearer understanding of printability for more accurate future work. In a study on 3D printing of food components, Ma et al.^17^ highlight that the relationship between rheological properties and printability remains unclear due to the complexity and variability of the print components. Conversely, Elbadawi et al.^18^ and Mirzaei et al.^40^ use the term printability and compare rheological and biological parameters, even when they do not present a precise definition. The reviewed literature indicates both vagueness and controversy regarding the concept of printability. This is also summarized in Table 4.

As a starting point for the discussion, this study adopts Copus’ definition, as it effectively encapsulates the concept of printability as a conjunction of multiple parameters.

### 3.7 Correlation of Parameters in Additive Manufacturing for Large-Scale Production and Normative Approaches

One approach to understanding parameters related to printability and large-scale production is to examine standardized industrial processes and safety regulations established by recognized organizations and agencies.^41^ The primary reference for standardization is the International Organization for Standardization (ISO), a nongovernmental organization that maintains an extensive database of tests and protocols designed to ensure the quality and safety of production processes. Additionally, the American Society for Testing and Materials (ASTM) provides critical standards for material testing and evaluation. Beyond these nongovernmental organizations, individual countries often supplement international standards with regulations from governmental agencies. In the context of bioinks, which contain biological components, regulatory bodies such as the U.S. Food and Drug Administration (FDA)^42^ and the Brazilian Health Regulatory Agency (ANVISA)^43^ contribute to establishing guidelines for the normative production of materials in compliance with regional legislation. These agencies frequently work together to development standardized protocols and testing methods, ensuring the validation of scientific research. Furthermore, established norms undergo periodic review to account for advancements in technology, economic factors, and societal needs.^44^

Several examples illustrate this standardization process. The ISO 17296-3:2014 standard establishes general principles for testing and characterizing raw materials, while the ISO/ASTM 52921:2013 standard provides terminology for additive manufacturing processes. Additionally, in December 2017, the FDA introduced technical considerations for manufactured medical devices, including standards for evaluating the use of additive manufacturing in biocompatibility testing. These standardized tests and protocols encompass a wide range of procedures, from evaluating the rheological properties of materials, such as ideal viscosity, to postprocessing techniques in manufacturing. This highlights the critical role of standardization in advancing research and production in the field of bioinks.^41^

With the increasing presence of additive manufacturing in research and industrial applications, discussions regarding the review and refinement of existing standards have gained prominence in the literature. As noted by García-Domínguez et al.,^45^ there are still gaps and uncertainties in normative procedures, particularly concerning polymer-based materials and bioinks. The recent surge in the use of these materials has led to the production of numerous items outside traditional industrial settings, creating regulatory challenges. Furthermore, the presence of biological components, such as living cells, can significantly alter the standard material parameters, necessitating further adaptation of testing protocols. Another critical aspect to consider is the shape and structural integrity of printed materials. Cell properties and the ability of printed objects to maintain structural stability can introduce significant variability, potentially affecting research outcomes. These challenges reflect the complexities involved in standardizing additive manufacturing processes.^41,44^

The concept of printability can serve as a framework to address these discussions. If properly defined, printability could encompass the entire scope of additive manufacturing processes, providing a more comprehensive approach to standardization in the field and doing so, it could contribute to increase standards normative such as ASTM F3659-24 which includes terminologies for bioprinting but does not establish a formal definition for printability.^46,47^

## 4 Results and Discussion

### 4.1 Printability Related to Scaffold Dimension Geometric Parameters Only

Since 2011, the term ″printability″ has increasingly appeared in studies focused on the feasibility of 3D printing scaffolds, despite the lack of a specific definition. For instance, Butscher et al.^48^ and Zhou et al.^49^ investigated the printability of calcium phosphate, where the term referred primarily to the powder’s flowability during extrusion, without direct consideration of shape fidelity/accuracy or cellular viability. Nevertheless, it is evident that the term has already been associated with the rheological properties of the material (Table 5).

**Table 5:** References of Studies Reviewed According to the Definition and Use of the Term “Printability”

term	references
printability related to scaffold dimensions only	Butscher et al. (2012); Zhou et al. (2014); Naghieh et al. (2021); Reaksame et al. (2021); He et al. (2016); Gorroñogoitia et al. (2022);
printability separated from shape fidelity	Yan et al. (2012); Zhang et al. (2017); Janmelenki et al. (2020); Chen et al. (2023);^59^
printability as shape accuracy only	Cleymand et al. (2021);Kyle et al. (2017); Alonso et al. (2020);^60^ Lee et al. (2016);^61^ Klar et al. (2019); Nelson et al. (2021); Lee et al. (2019)
printability as shape fidelity, but which takes cell viability as an essential item for bioink	Elbdawi et al. (2020); Chung et al. (2013);^62^ Ji et al. (2017);^63^ Ouyang et al. (2016).; Joungprasitkul et al. (2022); Karamchamd et al. (2022); Scalzone et al.[Bibr ref64] (2022); Bai et al. (2022);^65^ Coskun et al. (2022);^66^ Butler et al. (2021);^67^ Jia et al. (2014);^68^ Markstedt (2015);^69^ Copus et al. (2022); Suntonnord et al. (2022); Gao et al. (2018); Diamantides et al. (2017);^70^ Masri et al. (2021); Schwab et al. (2020); Feng et al. (2022);^71^ Lim et al. (2021);^72^ Soltan et al. (2019);^73^ Yin et al. (2019); Fudos et al. (2021);^74^ Karvinen. (2023);^75^ Kim et al. (2020);^76^ Rau et al. (2023);^77^ Rau et al. (2023b);^78^ Kim et al. (2019);^79^ Li et al. (2022)
printability that takes cell viability into account	Fu et al. (2021);^80^ Lee et al. (2020); Zhao et al. (2015); Clegg et al. (2019); Holzl et al. (2016);^81^ Kim et al. (2017);^82^ Thakare et al. (2022); Paxton et al. (2017); Luo et al. (2020);^83^ Chen et al. (2018); Lechner et al. (2022);^84^ Cooke et al. (2021);^85^ Habib et al. (2022);^86^ Wang et al. (2023).^87^

Similarly, Gorroñogoitia et al.^37^ emphasize shape fidelity as a key factor in assessing the printability of alginate polymers, reflecting the current approach where printability is often associated solely with the shape of scaffolds. This focus on structural integrity may be tied to the objective of creating materials that serve as frameworks for bone tissue prostheses, often without accounting for parameters such as cellular viability.

Additionally, He et al.,^50^ Naghieh et al.,^5^ and Reaksame et al.^51^ compare the printability of various bioinks and scaffolds, yielding insights that could enhance our understanding of printability. However, as highlighted in this study, for additive manufacturing—especially those employing data science—the consideration of additional parameters in bioinks may be necessary. Addressing ambiguities and avoiding vague definitions could significantly improve the quality of these studies and their feasibility.

Several studies have also considered shape fidelity as a critical factor in defining printability. This consideration is primarily attributed to the structural characteristics of the bioink components. For instance, as observed in the works of Klar et al.^35^ and Nelson et al.,^52^ bioinks with high aqueous content require precise shape maintenance after the printing process to ensure proper functionality. The perception of printability as distinct from form fidelity is often associated with printing methods that do not involve extrusion, such as laser-assisted printing. This technique is well-known for its sophisticated and high-resolution layer-by-layer printing capabilities with higher shape accuracy and shape fidelity.

For example, Yan et al.^53^ evaluated the printability of glycerol gels by measuring dimensionless droplet factors in relation to viscosity and density during capillary jet formation. Their findings guided the selection of gels based on glycerol concentrations in the mixtures.

Similarly, Zhang et al.^23^ utilized this approach to define the printability of bioinks containing cells—such as fibroblasts, bovine cells, and rat cells—incorporated into alginate gels. They observed that the inclusion of these cellular components affected printability during jet formation. Notably, their study also explored the relationship between cellular viability postprinting and laser intensity, with gel cell density being a key parameter.

This trend is further exemplified by studies like those of Janmelenki et al.^101^ and Chen et al.,^59^ which assess the printability of bioinks through gel formation. Despite its advantages, laser-assisted printing remains limited by its high cost and complexity, leading many research groups to rely on more conventional bioprinting methods.

### 4.2 Printability Separated from Cell Viability

The importance of cellular viability in bioinks has been recognized in the literature. However, a clear correlation between cellular viability and printability, or even the inclusion of this aspect in the definition, is usually neglected. Many studies treat cellular viability as a separate factor from printability, likely because viability assessments are typically conducted postprinting.

For instance, Karamchand et al.^54^ explore bioinks aimed at mimicking lung tissue, noting that mechanical properties and biocompatibility—termed ″cytohability″ in their study—are critical for bioink performance. Nevertheless, these parameters are still considered independently of printability.

In studies where printability is discussed separately from viability, the material type often plays a significant role. For example, Reaksame et al.^51^ examined the printability of alginate dialdehyde-gelatin bioinks with added methylcellulose to enhance printability. Their findings, which focus on scaffold shape fidelity, do not integrate cellular viability as part of the printability assessment. Similar approaches are observed in works involving hydrogels, such as those by Lee et al.^15^ and Suntornnond et al.,^55^ where cellular viability is treated independently of printability. Schwab et al.^10^ emphasize that while cellular viability, rheological properties, and shape fidelity are crucial for bioink manufacturing, printability remains a distinct concept from these factors.

This lack of consensus about the inclusion of the biological performance in the definition may hinder the progress of additive manufacturing research involving bioinks.

### 4.3 Printability: An Approach from Rheology, Shape Fidelity, Shape Accuracy, and Cell Viability

As expected, consensus exists in much of the reviewed literature regarding the necessity of understanding the interplay between the mechanical and rheological properties of bioinks, shape fidelity/accuracy, and cellular viability, both during and after printing. The complexity inherent in the printing process renders printability a highly relevant and multidisciplinary research area. An approach to printability that integrates these parameters is aligned with contemporary research demands. This section discusses articles that adopt such a comprehensive definition of printability.

Notably, Zhao et al.^56^ provided a profound analysis of printability in the context of 3D bioprinting, emphasizing that cellular viability is closely related to printability beyond merely assessing the mechanical properties of bioinks containing tissue culture cells. Their study demonstrated that evaluating printability requires not only measuring shape accuracy and shape fidelity but also assessing cellular survival rates. The study found that achieving satisfactory results across these parameters necessitates careful control of factors such as temperature and extrusion force during printing. Cleeg et al.^57^ addressed the challenges of using bioinks for tissue replacement and clinical testing, reflecting a focus on cellular viability. Additionally, research incorporating machine learning for printing modeling, such as that by Mirzaei et al.^40^ also seeks to connect printability with cellular viability. This approach may stem from the need for a comprehensive and dynamic model that better represents the bioprinting process with biological components. Although Thakare et al.^27^ do not explicitly define printability, their study of alginate-methylcellulose-GELMA bioinks indicates an implicit relationship between shape fidelity/shape accuracy and cellular viability, beyond rheological parameters. Their experimental results show correlations between changes in rheological properties, geometric parameters, and cellular viability, assessed through cellular density postprinting. Similarly, Jongprasitkul et al.^58^ explored the printability of methacrylated gellan gums, emphasizing the need to optimize both shape fidelity and cellular viability for improved printing outcomes. Despite these advancements, defining printability remains challenging and lacks consensus, impeding the precise characterization of the term. The inclusion of cellular viability in a broad definition of printability should emerge as a particular case when dealing with 3D printing involving bioinks or live cells.

### 4.4 Proposition for a Definition of Printability

Figure 3 presents a conceptual map illustrating that printability is inherently linked to groups of parameters encompassing geometric attributes of the print, as well as rheological properties and cell viability considerations. Additionally, it highlights the strategies employed to describe these parameters and Figure. Figure 4 provides an overview of the extrusion-based printing process and correlates the factors discussed herein with the concept of printability.

**Figure 3:**
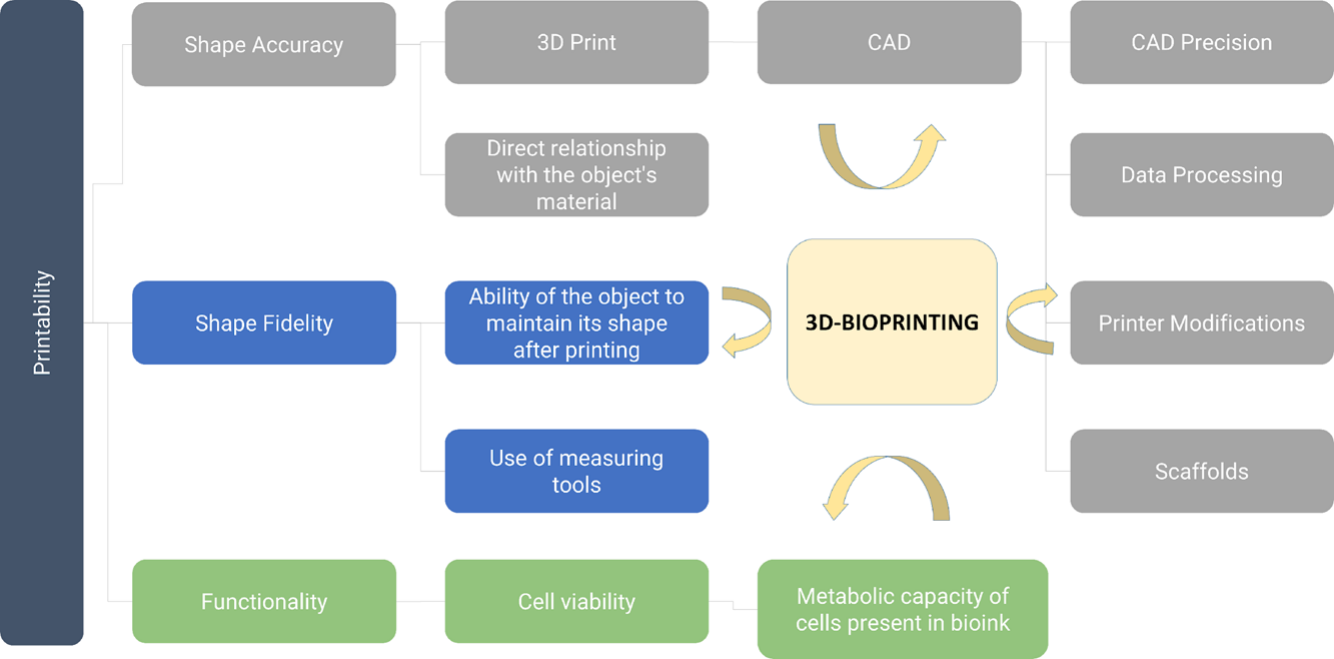
Representation of a conceptual map on printability and its definition parameters. It is noted that, along with the presented concepts, 3D bioprinting is correlated with the processes of object formation.

**Figure 4:**
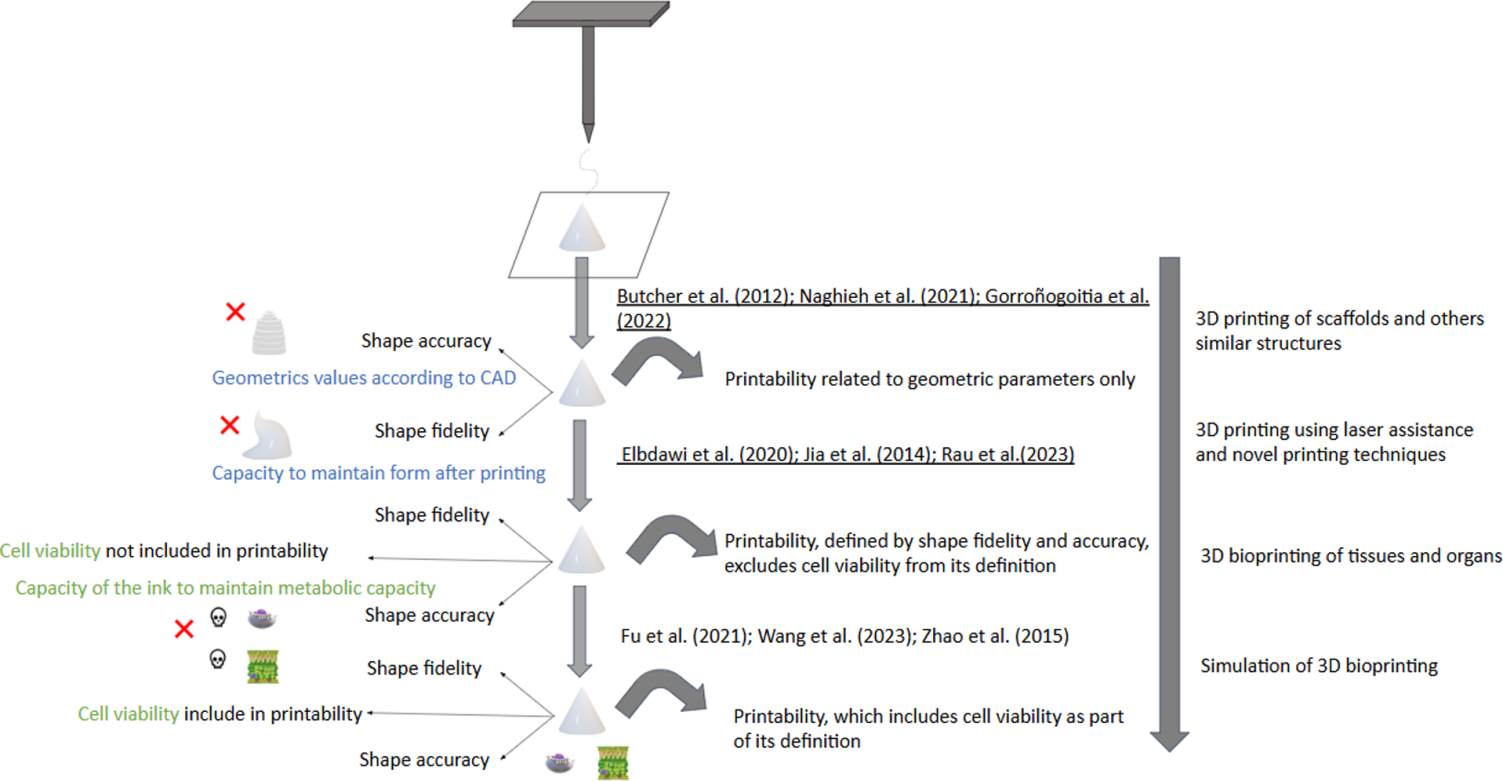
Representation of an ideal 3D bioprinting model with the printability parameters presented in the review.

Based on the discussion presented above, a reformulated definition of printability is proposed as follows: ″Printability is a measure of the ability to fabricate a 3D object using additive manufacturing with fidelity regarding a digitally designed prototype, in such a way that the geometrical characteristics (shape), as well as the functionality of the manufactured object, are preserved during and after a number of viable processing steps.″ The conceptualization of this definition is presented in Figure 2b indicating the key aspects that a broad definition must include to be valid not only for bioprinting.

Looking ahead, it is expected that future research will increasingly focus on correlating those parameters of bioprinting with printability. This work proposes a model that incorporates printability as a crucial factor in the printing of bioinks. It is expected that this literature review may contribute to defining printability with broader acceptance and to establishing eventual technical standards.

## 5 Conclusions

Over the years, it is noted that the field of 3D bioprinting has become an important area of innovation and discussion due to its interdisciplinary nature and broad applications in regenerative medicine and tissue engineering. The significance of this field is reflected in the increasing investments and number of published papers devoted to this topic. From 2014 to 2022, according to Grand View’s data, there has been approximately a 30% increase in global financial investment in this area. Several studies attribute this rise to the current organ transplant waiting list situation, coupled with a growing interest in the study of tissue bioprinting, as traditional organ acquisition methods often involve high costs and significant time.

However, as the search for alternative manufacturing methods intensifies, researchers face various challenges in establishing reliable methodologies for the fabrication process. Issues such as balancing rheological properties, aseptic/sterilization methods for bioprinting inks, postprinting shape fidelity, shape accuracy, and cellular viability of the material are commonly addressed. All these components can be linked to a single term: printability. As seen in this study, despite being cited repeatedly, printability exhibits some inconsistencies in its formal definition. We demonstrated that this scenario may arise due to the lack of a formal, including normative, definition for this term, making difficult the correlation of material characteristics with the additive manufacturing process of bioprinting. Another challenge highlighted by this review is the need for a biological perspective on bioprinting ink production. This implies the need for a model that considers not only strictly mechanical parameters but also the entire biological functional apparatus of cells. In the absence of cells, it is crucial to indicate whether the physiological or metabolic functionality of the bioprinting ink is effective.

Despite the extensive studies and findings on printability, a universally accepted definition has not been established in the literature yet. A tentative formal definition is proposed as a starting point within the bioprinting community in order to achieve a universally accepted definition.

This study has pointed out that the absence of an accepted formal definition of printability might impact the studies on printability in the field of bioprinting.

A viable approach to mitigate the challenges due to the complexity of obtaining suitable products by 3D bioprinting could involve the creation of mathematical and computational models for bioprinting simulation. Some studies have already adopted this approach, with recent ones competently verifying the extrusion of bioprinting inks, potentially preventing the waste of time and materials in research. Many of these models rely on constantly changing and expanding databases, directing the use of artificial intelligence and machine learning technologies to create them. However, the lack of a clearly defined object function (printability), means all those efforts can be useless.

## §

Interunit Graduate Program in Bioengineering (PPGIB), University of São Paulo, Avenida Trabalhador são-carlense, 400 Pq Arnold Schimidt, 13566-590 São Carlos, SP, Brazil. Tel.: +55 (16) 3373-9586

## ∥

Department of Basic Sciences of Faculty of Animal Science and Food Engineering (FZEA-ZAB), University of São Paulo, Rua Duque de Caxias, 225, 13635-900, Pirassununga, SP, Brazil. Tel.: +55 (19) 3565-4200.
